# Effective Approaches to Managing Behavioral Problems in Children With Attention-Deficit/Hyperactivity Disorder (ADHD): A Narrative Review

**DOI:** 10.7759/cureus.102011

**Published:** 2026-01-21

**Authors:** Ahmad Sughayyier Albalawi, Ahmed Ibrahim Khayyal, Fatimah Abdullah Alnasser, Nasser Naif M AlSuhaymi, Amjad Abdulaziz Mousa Assiri, Rehab Saud Saeed Alahmadi

**Affiliations:** 1 Pediatric Emergency Medicine, Maternity and Child Hospital, Tabuk, SAU; 2 Pediatrics, King Abdulaziz University Medical Services Center, Jeddah, SAU; 3 General Practice, Dar Al Uloom University, Riyadh, SAU; 4 General Practice, Al Rayan National College, Al-Madinah, SAU; 5 General Practice, Ministry of Health Holdings, Makkah, SAU; 6 General Practice, King Saud Medical City, Al-Madinah, SAU

**Keywords:** adhd, attention-deficit/hyperactivity disorder (adhd), behavioral disorder, children, management, pediatric

## Abstract

Attention-deficit/hyperactivity disorder (ADHD) is a neurodevelopmental disorder. It is considered one of the most common disorders in childhood. Its long-term adverse effects are associated with untreated or delayed ADHD in childhood. Impaired academic, functional, and social performance hinders a healthy life. Moreover, good and easy management of children is available via different approaches, providing better long-term improvement. This study aims to summarize the current interventional management strategies for ADHD collectively. We discuss the strengths and limitations of each intervention and highlight the existing gap. In addition, we discuss patient-centered and tailored approaches. This review includes the studies focusing on children and adolescents aged between three and 18. Management strategies include stimulant and non-stimulant medications, behavioral therapies, educational accommodations, lifestyle modifications, and complementary therapies such as mindfulness and yoga. Combining these interventions through a patient-centered approach improves outcomes. Timely diagnosis and early management reduce ADHD's adverse effects. The management plan for ADHD consists of behavioral therapy, educational or training therapy, pharmacological treatment, and lifestyle modification. Each line is effective alone; sometimes, we combine them to make a patient-centered and tailored approach.

## Introduction and background

Attention-deficit/hyperactivity disorder (ADHD) is a heterogeneous neurodevelopmental condition characterized by developmentally inappropriate patterns of inattention and/or hyperactivity-impulsivity that interfere with functioning across multiple settings [1,2]. Rather than representing a single, uniform disorder, ADHD encompasses a spectrum of symptom profiles, developmental trajectories, and functional impacts that vary by age, context, and comorbidity burden. Neurobiologically, ADHD has been associated with alterations in frontostriatal circuitry, neurotransmitter regulation, and executive functioning, contributing to variability in symptom expression and treatment response [3].

ADHD is commonly diagnosed in childhood, with prevalence estimates of approximately 7.6% among children aged 3-12 years and 5.6% among adolescents aged 12-18 years [3]. Symptom presentation evolves across development, with hyperactivity often more prominent in early childhood and inattention and executive dysfunction becoming more salient during adolescence. The impact of ADHD extends beyond core symptoms, affecting academic performance, social functioning, emotional regulation, and family dynamics. However, outcomes are highly variable, and many individuals experience meaningful improvement with appropriate recognition and support [4].

Early identification and evidence-based intervention are associated with improved functional outcomes and reduced risk of adverse sequelae, including academic underachievement and behavioral difficulties [4,5]. Conversely, delayed recognition or suboptimal management may contribute to persistent impairments in educational attainment, occupational functioning, and psychosocial well-being in a subset of individuals [6]. Importantly, not all children with ADHD experience severe long-term consequences, underscoring the need for balanced, individualized approaches that reflect the disorder's heterogeneity rather than worst-case trajectories [7].

Management of ADHD is inherently complex and extends beyond symptom reduction alone. Available interventions include pharmacological treatments (stimulant and non-stimulant medications) and non-pharmacological strategies such as behavioral therapy, parent training, educational accommodations, and lifestyle interventions [7,8]. While clinical guidelines support multimodal care, real-world decision-making is often challenged by age-specific considerations, comorbid conditions, family preferences, variability in treatment response, and differences in healthcare access and resources [9,10]. These factors contribute to ongoing debate regarding optimal treatment sequencing, integration of interventions, and long-term management strategies [11].

Despite a large and growing body of literature, existing reviews frequently examine pharmacological and non-pharmacological interventions in isolation or focus narrowly on efficacy outcomes without sufficient attention to clinical context and implementation. There remains a need for an updated narrative synthesis that integrates contemporary evidence across treatment modalities and frames ADHD management within a patient-centered and tailored care model. In this review, patient-centered care is defined as individualized treatment planning that accounts for developmental stage, symptom domains, functional priorities, family and educational context, and patient and caregiver preferences [12-14].

Accordingly, this narrative review aims to provide clinicians and allied healthcare professionals with a clinically oriented synthesis of current evidence on ADHD management, highlighting the challenges of real-world practice and emphasizing an integrated, individualized approach to care in children and adolescents with ADHD.

## Review

Methods

This study was conducted as a narrative review aimed at synthesizing and critically discussing current evidence on ADHD, with a focus on epidemiology, risk factors, clinical outcomes, and management strategies at any age. The review was not designed as a systematic or scoping review and therefore does not fully adhere to Preferred Reporting Items for Systematic Reviews and Meta-Analyses (PRISMA) or PRISMA-Scoping Reviews (ScR) reporting standards.

A comprehensive literature search was performed using PubMed, Web of Science, Scopus, and Embase. The search covered publications from January 2010 to April 2025, without language restrictions. Search terms were selected to capture a broad range of relevant literature and included combinations of the following keywords: "ADHD", "attention-deficit/hyperactivity disorder", "hyperkinetic disorder", "epidemiology", "risk factors", "behavioral therapy", "educational interventions", "pharmacological treatment", "lifestyle interventions", "complementary and alternative therapies", "family support", and "methodological limitations". Reference lists of key articles and relevant reviews were also screened to identify additional pertinent studies.

Studies were considered eligible if they addressed ADHD in children and adolescents, including topics related to prevalence, risk factors, clinical outcomes, and management strategies. Priority was given to clinical trials, observational studies, meta-analyses, umbrella reviews, and clinical guidelines relevant to pediatric ADHD. 

Titles and abstracts were screened for relevance, followed by full-text reviews of potentially eligible articles. Information extracted from selected studies. Study selection and data extraction were conducted to support a structured narrative synthesis rather than quantitative pooling of results.

Evidence was synthesized using a qualitative narrative approach, organizing findings thematically across epidemiology, risk factors, clinical outcomes, and intervention modalities. Emphasis was placed on identifying patterns, areas of consensus and controversy, and gaps in the existing literature. Greater weight was given to high-quality evidence, including meta-analyses, umbrella reviews, and clinical practice guidelines, while also acknowledging methodological limitations and heterogeneity across studies.

Consistent with narrative review methodology, no formal risk-of-bias assessment or quantitative meta-analysis was performed. This approach was chosen to allow flexible interpretation of heterogeneous evidence and to avoid over-standardization of diverse study designs. The absence of formal bias assessment and statistical synthesis has been explicitly stated to prevent misinterpretation of the review's methodological scope.

Prevalence and epidemiology of ADHD

ADHD's estimated global prevalence is 8% among children and adolescents. ADHD has three types: inattentive, hyperactive, and combined. The inattentive type (ADHD-I) is the most prevalent type [15].

The prevalence of ADHD among children in the United States is estimated at 11.4%, which is comparable to rates reported in the Middle East and North Africa (MENA) region (10.1%) but higher than in Europe, where prevalence generally ranges from 4% to 7% [16-18]. These regional differences have been attributed to variations in diagnostic criteria, study methodologies, cultural perceptions of behavioral disorders, and disparities in healthcare access and utilization.

Prevalence also varies by age: 2.4% among young children aged 3-5, 11.5% among school-age children aged 6-11, and 15.5% among adolescents aged 12-17 [17,19].

ADHD prevalence varies according to demographics, such as gender, race, and ethnicity. In the U.S., ADHD is more prevalent among boys (15%) than girls (8%). The prevalence also varies throughout different races. The prevalence among Black and White children is 16%, while Asian children have a lower rate (4%). The prevalence among American Indian/Alaska Native children is 10%, and 6% among Native Hawaiian/Pacific Islander children. Additionally, it is more prevalent among non-Hispanic children (12%) than Hispanic children (10%) [19,20].

Impact of ADHD on children's health and functioning

ADHD has a negative adverse effect on pediatrics. These effects include the persistence of ADHD through time, academic adverse effects, occupational adverse effects, and physical and mental adverse effects.

Persistence of ADHD

ADHD is a chronic condition. It persists from childhood till adulthood. Most of the longitudinal studies that studied ADHD in pediatrics showed persistent symptoms in a substantial percentage of the diagnosed children throughout childhood. According to Barbaresi et al., one-third of the children diagnosed with ADHD continued to experience it around age 27 [21]. Similarly, Biedermen et al. showed a high persistent rate of 35% in their follow-up study, and 6% showed remission but were still on active treatment [22]. According to Owens et al., ADHD showed a persistent rate of 50%-80% into adolescence and a persistent rate of 35% into adulthood during a follow-up study done in 2015 [23]. Other longitudinal studies highlighted a significant persistent rate: the Montreal Study found that 66% of hyperactive ADHD patients showed persistent symptoms in adulthood. The Milwaukee Study reported a 14-35% recovery rate at age 27. The Multimodal Treatment Study of Children with ADHD (MTA) showed a persistent rate of 49.9% at the age of 25 [24]. This highlights the lifelong nature of this disorder among boys and girls, even after passing childhood. They still show symptoms and suffer from ADHD.

Educational and Academic Functioning

Children with early diagnosis of ADHD show significant academic failure compared to their non-ADHD peers. This academic impairment is assessed as passed-on grades, academic achievements, school expulsion, repeating grades, and college dropout rate [24]. The previous indicators collectively measure the academic adverse effects associated with ADHD. Additionally, this academic impairment extends from childhood to adulthood. Thus, it significantly affects the financial level [24]. This academic impairment affected approximately 413,000 to 1.65 million college students with ADHD in the U.S. in 2012, with a percentage of 2% to 8 %, according to The National Center for Education Statistics [25].

The Montreal study proved that children with ADHD had poor academic outcomes affecting all indicators. Similarly, half of the participants with ADHD in the Massachusetts General Hospital (MGH) study had below "C" grades frequently after having special classes and repeating each class at least twice. Additionally, children with ADHD in the Pittsburgh ADHD Longitudinal Study (PALS) showed lower school GPAs. According to the studies, 5%-17% of participants with ADHD can hold a bachelor's degree compared to 30% of the control group. Thirty-two percent of children with combined ADHD dropped out of high school and could not make it to college, compared to 15% of adults without any psychiatric disorder [24].

According to Kuriyan et al., ADHD participants were 11 times less likely to demonstrate discipline in a four-year college. On the other hand, only 15% of participants with ADHD, when compared to the control group, could earn a four-year bachelor's degree vs. 5.4% of the control group [26].

Occupational Functioning

This adverse effect appears in young adults aged from 23 to 32 years. It is outside the age range of pediatrics. However, it is a consequence of childhood ADHD [26]. Kuriyan et al. showed that participants with ADHD were 11 times more likely to be unemployed. Sixty-one percent of workers with ADHD suffered from being fired. Additionally, 53% were likely to quit their job due to dislike. Kuriyan et al. also highlighted that participants with ADHD could not earn more than two USD per hour and a loss of up to $1.27 million [26].

On the other hand, the Montreal study divided participants with ADHD according to their age group into two subgroups: young adulthood aged (17-24 years) and the other group (>24 years). The young adulthood group shows no difference in work performance compared to participants without ADHD. While in the age group (17-24 years), participants with ADHD quit their jobs frequently compared to the control group [24,26]. They also showed poor performance assessments when rated by employers. Multiple longitudinal studies, including the New York Study, the Milwaukee Study, the Massachusetts General Hospital (MGH) Study, the Pittsburgh ADHD Longitudinal Study (PALS), the Berkeley Girls with ADHD Longitudinal Study (BGALS), and the Multimodal Treatment Study of Children with ADHD (MTA), have consistently shown that individuals diagnosed with ADHD in childhood experience higher rates of work instability and financial dependence, as well as lower socioeconomic status, in adulthood [24].

Mental Health

Similar to occupational adverse effects, childhood ADHD has serious long-term mental health consequences in adulthood. ADHD participants in the Montreal study showed high suicidal attempts and multiple complicated psychiatric diagnoses compared to the control group. Besides, participants with ADHD in the Milwaukee Study were four times more likely to have major depression, depressive personality, and dysthymia, and five times more likely to have generalized anxiety disorder. The MGH study showed a strong association between ADHD and comorbid mood. At least 84% of ADHD participants had comorbid disorders, anxiety disorders, emotional dysregulation, autistic traits, and traumatic brain injuries. Suicidal attempts are the most dangerous mental health side effects of all. Notably, 22% of girls with combined ADHD and 7.7% with inattentive ADHD had attempted suicide [24].

Physical Health

The Milwaukee Study used 14 indicators to assess physical health. One of these indicators was life expectancy. Children with ADHD were more likely to show a reduction in life expectancy by 10 years. Additionally, adulthood ADHD was more likely to show a life expectancy reduction by 13 years [27]. Additionally, participants with ADHD were more likely to have emergency admission, traumatic head injuries, and an increased mortality rate than participants without ADHD [24,28].

Substance Use

Multiple longitudinal studies found an increased addiction rate among adult ADHD, especially the hyperactive subtype [24,29]. About 50% of ADHD participants show substance abuse disorders [30,31].

Low self-esteem and social and interpersonal difficulties are widely common adverse effects of ADHD. They are consequences of dysregulation and impulsivity. Notably, financial burden is a core adverse effect of ADHD. It results from multiple arrests, job quitting, and poor work and academic performance. Impaired family relationships and loneliness are prevalent among those with ADHD. Additionally, females with ADHD are more likely to suffer from these social adverse effects than males. Thus, females with ADHD are more likely to show internalizing disorders [24,31].

Risk factors for ADHD

ADHD is a neurodevelopmental disorder, in line with DSM-5 terminology. We can categorize the likelihood of risk factors to increase ADHD prevalence into prenatal, child-related, family-related, and environmental risk factors (Table 1).

**Table 1:** ADHD risk factors ADHD - attention-deficit/hyperactivity disorder Sources: [24,29-31]

Prenatal	Child-related	Family-related	Environmental
Stress and anxiety, alcohol exposure, tobacco exposure, cannabis exposure, cocaine exposure, mercury exposure, antidepressant exposure, pregnancy complications	No breastfeeding, neonatal illness, childhood infection, head injury, sleep problem	Child media exposure, tobacco exposure, parenting incapacity, abuse, negative discipline, divorce, single-parenting	Lead exposure, organophosphates, mercury exposure, polychlorinated biphenyl exposure

Prenatal Risk Factors

Bitsko et al., Maher et al., and Robinson et al. studied the association between multiple risk factors during the prenatal period and the risk of delivering a baby with ADHD [32-35]. There were significant associations between maternal tobacco and alcohol use, first-trimester antidepressant use, and later-onset offspring diagnosis with ADHD [35]. Bitsko et al. found a strong association between preterm birth and ADHD, with an odds ratio (OR) of 8.68 for ADHD in preterm infants compared to term infants [33].

Child-Related Risk Factors

Child health has a strong association with ADHD. Increased birth weight is strongly associated with increased odds of ADHD (OR=3.36) [33,34]. There are multiple risk factors associated with the likelihood of ADHD, such as neonatal disease, absence of breastfeeding, sleep disturbances, head injury, and early childhood infections [32,33].

Family-Related Risk Factors

The family has a significant effect on the risk of offspring developing ADHD. Offspring from socially separated families showed a lower threshold for developing ADHD [32]. Claussen et al. found a strong association between parental psychopathology, including depression, anxiety, antisocial personality disorder, substance use disorders, and ADHD in offspring [36]. Family abuse is prevalent in 9-15% of children with ADHD symptoms [32-36].

Environmental Risk Factors

Exposure to environmental toxins such as lead and organophosphates is associated with an increased risk of ADHD. Similarly, increased screen time in childhood increases the risk of later diagnosis with ADHD. Notably, delayed diagnosis and intervention lead to exacerbating adverse effects [37].

Different interventions in the management of ADHD

Behavioral Therapy Interventions

Behavioral interventions depend on changing behavioral contingencies. Thus, they increase a child's willingness to do the desired actions and self-limit the undesirable. They include parent training, classroom interventions, and peer-based interventions. They enable parents and caregivers to deepen their understanding and enhance parenting modalities [38,39]. Behavioral intervention tends to teach caregivers to catch undesirable actions by children and oppose them. Additionally, they encourage the child by giving positive attention and praise when they notice desirable action [38,40]. 

Parent training: 7-12 sessions of parent training behavioral management (PTBM) are delivered to parents and caregivers about interventions. The structured PTBM approaches are described in Table 2 [41].

**Table 2:** PTBM approaches PTBM - parent training behavioral management; Triple P - Positive Parenting Program; PCIT - Parent-Child Interaction Therapy; NFPP - New Forest Parenting Program Sources: [38-41]

PTBM approaches	Brief description	Age range
Triple P	It aims to prevent and treat behavioral and emotional problems in children and adolescents by equipping them with skills to be self-sufficient and provide ongoing support to manage family issues.	Birth 0-12 years; teen triple P: 12-16 years
PCIT	It is an evidence-based treatment when a therapist watches ongoing sessions with the parent and child in an observation room and provides coaching skills in real time through a behind-the-ear device.	2-8 years
Incredible years	It is a set of DVDs based on interlocking and comprehensive programs that target parents, children, and teachers, where trained facilitators use video vignettes to structure and stimulate group discussions, problem-solving, and trigger practices.	Parent-based: 0-12 years; teacher-based: 3-8 years
Kazdin method	It helps with parenting strategies to handle behavioral problems by developing specific behaviors they want and developing positive traits like kindness, honesty, respect, etc., in a nurturing environment at home.	6-12 years
NFPP	It is a parenting program for moderate to severe ADHD and takes place in family homes through eight weekly visits where parents are made aware of symptoms and signs of ADHD, and parents learn strategies to manage a child's behavior and attention difficulties.	3-11 years

Classroom or school-based interventions: ADHD children may need extra classes and specialized educational services. In 1999, the government listed ADHD patients in the Individuals with Disabilities Education Act (IDEA) [42]. A group uses a two-pronged test to determine if a child needs special education services. These two people assess whether this disability (ADHD in this case) influences educational functioning. Thus, an individualized education program (IEP) is prepared, applied, and monitored by trained teachers [43]. If a child does not show improvement with the initial intervention, escalation to the next recommended treatment step is considered. It is Section 504 of the Rehabilitation Act of 1973 that provides specific facilities to the child, like providing a quiet place, homework reduction, clear direction for homework, dividing tests into small multiple tests, modifying test format, and increasing time for test completion [44]. According to Charach et al., academic performance improvement is related to the intervention period [45].

Peer-based interventions: These interventions are group-based. Notably, more than 50% of children with ADHD faced problems with their peers [38]. Thus, working on quality friendships is crucial. This intervention has three subtypes [46]. The first is about peers facilitating each other's learning process via interactions such as helping and praising. The second is called peer-mediated intervention, where a peer is trained to deliver instructions to their peers with ADHD that lead to improvement of social skills. The third is peer proximity intervention, where a highly skilled child is seated at the same table with a child with ADHD. The structured peer-mediated interventions were used and were effective in improving peer relationships without adjunctive pharmacological treatment [46]​​​​​.

Educational and Training Interventions

Cognitive Behavioral Therapy (CBT): This intervention focuses on relieving ADHD symptoms, so the children become more effective. Additionally, CBT is useful for depression and other mental disorders [47]. Boyer et al. demonstrated that the planning skills the child develops through CBT improve symptoms [48].

Cognitive training: This training has a significant effect on memory. There are Cogmed Working Memory Training (CWMT) and Braingame modality [49]. For example, using the game "Braingame Brain" showed significant improvement in working memory [50,51].

Biofeedback and neurofeedback: This technique enables children with ADHD to control their heart rate and breathing. The child is connected to sensors that send this information to the computer [38]. Thus, children often consider this intervention a computer game [51]. This intervention changes a child's brain activity visually. This modality enhances the neural network that needs to be focused on, improving executive function [52].

Organizational skills training: This intervention aims to build organization, cooperation, and time management skills in children with ADHD. Children use daily planners, to-do lists, or tasks that are broken into parts. Bul et al. designed Plan-it Commander, which enabled children to manage their time properly and was highly rated by teachers [53].

Pharmacological Interventions

The drugs are classified as stimulants and non-stimulants.

Stimulants: They are the first-line drugs for managing ADHD due to the extensive evidence of efficacy and a known safety profile. There are two types of stimulants: amphetamine-based and methylphenidate-based [54-58] (see Tables 3-4).

**Table 3:** Amphetamine-based stimulants for ADHD treatment ADHD - attention-deficit/hyperactivity disorder; IR - immediate-release; XR - extended-release Sources: [54-58]

Medication name	Formulation	Duration of action	Unique features	Common side effects	Special considerations
Amphetamine mixed salts	Immediate-release, extended-release	IR: 4-6 hours, XR: 10-12 hours	Widely used; available in multiple generic options.	Insomnia, decreased appetite, weight loss, irritability	Assess risk of misuse or addiction. Counsel on proper storage and signs of overdose.
Dextroamphetamine	Immediate-release, solution	IR: 4-6 hours	Includes formulations like Xelstrym™, the first transdermal amphetamine patch (9-hour wear time).	Nervousness, headache, dry mouth, abdominal pain	Xelstrym™ requires rotation of application sites and caution with heat exposure as it increases drug absorption.
Lisdexamfetamine	Prodrug (capsule or chewable tablet)	10-12 hours	Prodrug formulation reduces misuse potential; converted to active drug in the GI tract.	Decreased appetite, fatigue, nausea, insomnia	Longer onset of action compared to other amphetamines; useful for patients at risk of misuse.
Amphetamine sulfate	Immediate-release	4-6 hours	Simple amphetamine formulation; available as a generic drug to reduce cost.	Increased heart rate, insomnia, anxiety	Short duration may require multiple daily doses, increasing the risk of adherence issues.
Amphetamine extended-release	Oral disintegrating tablet (ODT), suspension	10-12 hours	Includes formulations like Adzenys ER™ suspension, which combines immediate-release and delayed-release microparticles.	Agitation, dizziness, loss of appetite	Adzenys™ allows for suspension delivery, aiding patients unable to swallow tablets. Equivalent dosing to Adderall*®* XR.
Mydayis*®*	Extended-release (capsule with 3 delivery layers)	Up to 16 hours	Longest duration amphetamine formulation; combines immediate-release and two delayed-release beads.	Insomnia, decreased appetite, weight loss	Approved for adolescents (≥13 years) and adults only. Requires early morning administration due to long duration.

**Table 4:** Methylphenidate-based stimulants for ADHD treatment ADHD - attention-deficit/hyperactivity disorder; IR - immediate-release; XR - extended-release; SR - sustained-release; LA - long-acting; AAP - American Academy of Pediatrics;  NICE - National Institute for Health and Care Excellence Sources:  [54-58]

Medication name	Formulation	Duration of action	Unique features	Common side effects	Special considerations
Methylphenidate hydrochloride	Immediate-release, extended-release (LA, SR)	IR: 3-4 hours, LA/SR: 6-8 hours	First-line for younger children per AAP/NICE guidelines. Available as generic options.	Nervousness, decreased appetite, insomnia, headache	Monitor for misuse, addiction, and diversion. Counsel on proper storage and disposal of unused medication.
Methylphenidate extended-release	Osmotic-controlled release tablet (Concerta®)	10-12 hours	Concerta® uses an osmotic release mechanism; tablet shell may be visible in stool.	Dry mouth, dizziness, insomnia, nausea	Avoid in patients with severe GI narrowing. Not interchangeable with other methylphenidate formulations.
Dexmethylphenidate	Immediate-release, extended-release	IR: 4-6 hours, XR: 8-12 hours	Active isomer of methylphenidate; available in simpler dosing options.	Stomach pain, appetite suppression, irritability	Requires careful titration; effects on core symptoms are comparable to racemic methylphenidate.
Methylphenidate transdermal patch	Transdermal patch (Daytrana®)	9-12 hours	Only transdermal methylphenidate option; applied daily to the skin.	Skin irritation, delayed sleep onset, decreased appetite	Requires rotation of patch sites. The patch must be removed after 9 hours to avoid prolonged drug exposure.
Azstarys®	Prodrug capsule (serdexmethylphenidate/ dexmethylphenidate)	Up to 13 hours	First prodrug in the methylphenidate class; bioactivated in the lower GI tract for extended symptom control.	Insomnia, appetite suppression, headache	Capsule can be opened and mixed with food or water for easier administration.
Jornay PM®	Delayed-release/extended-release capsule	Up to 13 hours (administered at night)	Unique evening dosing to improve morning ADHD symptoms; designed for delayed morning symptom control.	Insomnia (common due to long duration), nausea, headache	Timing of evening dose is critical (6:30-9:30 PM). Not interchangeable with other methylphenidate products.
Adhansia XR™	Long-acting multilayer beaded capsule	Up to 16 hours	Longest acting methylphenidate formulation; designed for all-day symptom control.	Insomnia, dizziness, appetite suppression	Discontinued in July 2022 for business reasons, not safety concerns.

Non-stimulants

The shift to non-stimulants is done when symptoms don't respond to any other line of treatment (Table 5).

**Table 5:** Non-stimulant medications for ADHD treatment ADHD - attention-deficit/hyperactivity disorder; NRI - selective norepinephrine reuptake inhibitor; NDRI - norepinephrine-dopamine reuptake inhibitor Sources: [54,56,59]

Medication name	Mechanism of action	Key features	Common side effects	Special considerations
Atomoxetine	Selective NRI. Increases norepinephrine and dopamine in the prefrontal cortex.	Takes 1-2 weeks for initial benefit and 4-6 weeks for maximal effect. Available as a capsule in seven strengths (generic available).	Drowsiness, decreased appetite, nausea, fatigue, dry mouth	Boxed warning: suicidal ideation in children/adolescents. Monitor blood pressure and heart rate. Dose adjustments needed for CYP2D6 poor metabolizers.
Viloxazine extended-release	Selective NRI. Inhibits norepinephrine transporters in the prefrontal cortex.	Delivered once daily in capsule formulation. Takes 1-2 weeks for benefit and 4-6 weeks for maximal effect.	Drowsiness, irritability, nausea, fatigue	Boxed warning: suicidal ideation in children/adolescents. Strong CYP1A2 inhibitor. Avoid coadministration with sensitive CYP1A2 substrates.
Guanfacine extended-release	Alpha-2 adrenergic receptor agonist. Stimulates postsynaptic alpha-2 receptors to reduce hyperactivity and impulsivity.	FDA-approved for ADHD in children/adolescents. Extended-release formulation, dosed once daily in the morning.	Sedation, hypotension, bradycardia, dry mouth	Do not stop abruptly due to the risk of rebound hypertension. Monitor for repeated orthostatic symptoms or fainting; adjust dose if necessary.
Clonidine extended-release	Alpha-2 adrenergic receptor agonist. Stimulates postsynaptic alpha-2 receptors to manage ADHD symptoms.	FDA-approved for ADHD in children/adolescents. Extended-release formulation, dosed once daily in the morning.	Sedation, bradycardia, hypotension, dry mouth	Similar to guanfacine: avoid abrupt discontinuation due to rebound hypertension. Monitor for cardiovascular effects and sedative impact.
Bupropion	NDRI	Off-label for ADHD in adults and adolescents. Useful for patients with comorbid depression.	Insomnia, headache, dry mouth, increased heart rate	Should not be used in patients with a history of seizures or eating disorders. Monitor for mood changes.

Combination Therapy

A combination of stimulant, non-stimulant, educational, and behavioral interventions offers better control of symptoms and helps to overcome impairments such as emotional dysregulation.

Lifestyle and Home-Based Interventions

Lifestyle modification includes physical activity and a healthy diet. U.S. guidelines recommend one hour of daily physical activity for children aged six to 17 [60]. Physical activity helps with cognitive functions like memory and attention [60]. Pontifex et al. showed that aerobic exercise improved neurocognitive function [61]. Dietary recommendations for ADHD patients include avoiding fast food and artificial food coloring. Notably, Patrick et al. proved that vitamin D and omega-3 fatty acids help cognitive function and improve attention and behavior in ADHD patients [62]. 

Patient-Centered Care, Complementary, and Alternative Therapy

The National Center for Complementary and Integrative Health (NCCIH) describes integrative health care as a patient-focused, holistic approach to healthcare and wellness [63]. This approach, which values the individual and their unique needs, is particularly appealing to parents of children with ADHD. Almost 50% to 64% of parents report using integrative medicine to treat ADHD symptoms [64]. Interest in complementary approaches such as yoga, meditation, tai chi, and hypnotherapy has increased; however, the pediatric ADHD evidence base remains limited and methodologically heterogeneous. Many studies involve small samples, short follow-up periods, or extrapolation from adult populations [65].

Meditation is a powerful tool as it enhances attention to the present moment using focus and sustained attention. A 2018 meta-analysis of randomized controlled trials (RCTs) found that meditation-based therapies were associated with modest improvements in attention, behavioral regulation, and emotional functioning in children with ADHD, although the overall quality of evidence was moderate and results were heterogeneous [66]. This promising result offers hope for parents and professionals alike. 

Yoga helps children to build control over their mental and physical activity simultaneously. It offers strategies to increase attention and emotion regulation skills, which are core to ADHD [67]. A meta-analysis of eight RTCs suggests that aerobic exercise had a moderate to large effect on core symptoms such as attention (standardized mean differences, SMD=0.84), hyperactivity (SMD=0.56), and impulsivity (SMD=0.56), and related symptoms such as anxiety (SMD=0.66), executive function (SMD=0.58), and social disorders (SMD=0.59) in children with ADHD. Yoga exercise suggests an improvement in the core symptoms of ADHD. The main cumulative evidence indicates that short-term aerobic exercise, based on several aerobic intervention formats, seems to be effective for mitigating symptoms such as attention, hyperactivity, impulsivity, anxiety, executive function, and social disorders in children with ADHD [65].

Long-term practice of tai chi alone has been found to improve attention and reduce emotional outbursts, irritability, and anxiety in adults [68].

Clinical hypnotherapy involves the use of therapeutic suggestions delivered during a state of focused attention and altered consciousness to facilitate mind-body regulation, sharing conceptual similarities with other self-regulatory approaches discussed earlier in this review, such as mindfulness-based interventions and yoga [69]. There has been minimal research into hypnotherapy's impact on ADHD, particularly in children. Within the context of clinical hypnotherapy, susceptibility refers to an individual's responsiveness to hypnotic suggestion. Evidence suggests that children with ADHD who are treated with methylphenidate may demonstrate increased hypnotic suggestibility, potentially enhancing their responsiveness to hypnotherapeutic interventions when used as an adjunctive, non-pharmacological treatment [69]. Attention is one of the key factors in both hypnotic processes and patients with ADHD. In addition, the brain areas associated with hypnosis and ADHD overlap in many respects. However, the use of hypnosis in ADHD patients has still received only minor attention in research. There was a statistically significant decrease in reaction times in both the ADHD and control groups between the Continuous Performance Test (CPT) 2 and 3. The differences between CPT1 and CPT2, even though non-significant, were different in the two groups: in the ADHD group, reaction times decreased, whereas in the control group, they increased. Both groups made very few errors in the short CPT. Therefore, this study indicates that hypnotic suggestions have an effect on reaction times in the sustained attention task both in adult ADHD patients and control subjects. The theoretical and clinical implications are discussed [70].

Role of family and community support

Family and community share in the management plan for ADHD. Family-based interventions are very effective. Parent training and different approaches to PTBM showed robust efficacy in detecting and improving ADHD symptoms [71].

Moreover, Individualized Education Programs (IEPs) or 504 plans, teacher training, and classroom behavioral interventions are critical to implementing a comprehensive management plan for ADHD patients. School trips and activities, peer support, and mentorship reduce risk behaviors in children with ADHD [71].

Integration of clinical guidelines and hierarchy of evidence

Contemporary clinical management of ADHD is primarily guided by evidence-based recommendations from major professional bodies, including the American Academy of Pediatrics (AAP), the National Institute for Health and Care Excellence (NICE), and the Canadian ADHD Resource Alliance (CADDRA). These guidelines emphasize a stepped, multimodal approach that integrates pharmacological and non-pharmacological interventions according to age, symptom severity, functional impairment, and family context. While individual randomized trials provide important efficacy data, guideline-level syntheses offer a higher level of clinical relevance by incorporating comparative effectiveness, safety, feasibility, and real-world implementation considerations [72-74]. The AAP first published clinical recommendations for evaluation and diagnosis of pediatric ADHD in 2000; recommendations for treatment followed in 2001. The guidelines were revised in 2011 and published with an accompanying process of care algorithm (PoCA) providing discrete and manageable steps by which clinicians could fulfill the clinical guideline's recommendations. Since the release of the 2011 guideline, the Diagnostic and Statistical Manual of Mental Disorders has been revised to the fifth edition, and new ADHD-related research has been published. These publications do not support dramatic changes to the previous recommendations. Evidence is clear regarding the legitimacy of the diagnosis of ADHD and the appropriate diagnostic criteria and procedures required to establish a diagnosis, identify comorbid conditions, and effectively treat with both psychosocial and pharmacologic interventions. The steps required to sustain appropriate treatments and achieve successful long-term outcomes remain challenging [72].

Global Evidence and Contextual Variability

Despite ADHD being a globally prevalent condition, most high-quality intervention studies originate from high-income countries. Evidence from low- and middle-income countries (LMICs) remains sparse, particularly regarding non-pharmacological interventions and long-term outcomes [75]. Differences in diagnostic practices, healthcare infrastructure, educational systems, and cultural perceptions further limit generalizability. Between 1990 and 2021, global ADHD incidence, prevalence, and disability-adjusted life years (DALYs) increased by 9.92%, 18.71%, and 18.57%, respectively, with the highest burden among children and adolescents. Males consistently exhibited higher rates across all measures. High and high-middle socio-demographic index (SDI) regions experienced an increase in ADHD burden, whereas low SDI regions showed stable or declining trends. Bayesian age-period-cohort (BAPC) projections indicate continued growth in ADHD cases, with a particularly steep rise anticipated among females, indicating a potential narrowing of the historical gender gap in ADHD prevalence [75].

Implementation science and real-world clinical challenges

Clinical decision-making in ADHD extends beyond efficacy data and is shaped by treatment adherence, accessibility, caregiver burden, and sequencing of interventions over time. Implementation science studies indicate that real-world effectiveness often differs from trial-based efficacy due to resource constraints, clinician training variability, and family preferences [45]. These factors contribute to ongoing controversy regarding optimal treatment pathways and highlight the need for individualized, patient-centered care models. 

Limitations related to methodology and implementing interventions

Although behavioral and psychosocial interventions remain integral to ADHD care, evidence for certain complementary and cognitive-based therapies is mixed. Meta-analyses of cognitive training interventions consistently report improvements in trained tasks but limited transfer to core ADHD symptoms or everyday functioning [76]. Similarly, neurofeedback and mindfulness-based interventions show inconsistent effects, with several high-quality studies reporting null or modest benefits when rigorous controls are applied [77].

Additionally, many studies lack long-term follow-up, which limits understanding of the treatment effect. The outcome measure is based on parent and teacher notices, increasing bias risk. Families with ADHD may face challenges that hinder them from participating in interventions and structured programs. Thus, there is reduced engagement in interventions and less efficacy. Health system limitations include insufficient funds and a lack of psychological services. All the previous barriers undermine the continuity and coordination of care, reducing the overall effectiveness of interventions.

## Conclusions

Attention-deficit/hyperactivity disorder (ADHD) is a prevalent, multifactorial neurodevelopmental condition that substantially impairs academic achievement, occupational functioning, and social adaptation in affected children. Timely diagnosis and early, appropriate management are therefore critical to mitigating its long-term adverse consequences.

Effective ADHD management typically involves a multimodal approach encompassing behavioral interventions, educational and skills-based therapies, pharmacological treatment, and lifestyle modifications. While each modality may confer benefit when used independently, combining interventions within a patient-centered and tailored framework often yields superior functional outcomes. However, challenges to optimal ADHD management remain, particularly the heterogeneity of treatment responses and methodological limitations within the existing evidence base, highlighting the need for well-designed longitudinal studies and individualized, patient-centered treatment strategies.
